# Enhancing the Understanding and Utilization of Hypercoagulable Workup: A Quality Improvement Initiative

**DOI:** 10.7759/cureus.89004

**Published:** 2025-07-29

**Authors:** Colleen Bramwell, Keerthy Joseph, Yuan Liu, Pooja Rani, Abigail Sheridan, Parampreet Kaur, Nicole Agostino

**Affiliations:** 1 Internal Medicine, St. Luke’s University Health Network, Easton, USA; 2 Hematology and Medical Oncology, St. Luke’s Hospital, Easton, USA; 3 Hematology and Medical Oncology, St. Luke’s University Health Network, Easton, USA; 4 Research and Quality Improvement, St. Luke’s University Health Network, Easton, USA; 5 Hematology and Medical Oncology, St. Luke’s University Health Network, Bethlehem, USA

**Keywords:** electronic medical record, hypercoagulable, inappropriate test utilization, provider education, quality improvement

## Abstract

Background

In hospitalized patients, hypercoagulable testing is frequently overutilized, particularly in the context of acute thrombosis or active anticoagulation, when certain results may be unreliable and are unlikely to affect clinical management. This quality improvement (QI) initiative aimed to improve provider understanding and test utilization through electronic medical record (EMR) changes and targeted education.

Methodology

A retrospective chart review was performed across the St. Luke’s University Health Network’s campuses to assess thrombosis panel orders from January to April 2024. Following interventions that implemented EMR changes and improved provider education, a post-intervention review was conducted from December 2024 to April 2025. Orders were evaluated for appropriateness based on established guidelines.

Results

Of the 262 thrombosis panels ordered pre-intervention, 90.5% were deemed inappropriate, with only four identifying a true hypercoagulable condition. Following the intervention, appropriate ordering improved from 9.7% to 40.2%. There was a notable reduction in hypercoagulable workups ordered without a clear indication or confirmed venous thromboembolism, suggesting increased provider awareness of appropriate testing criteria. Inappropriate inpatient ordering of protein C, protein S, and/or antithrombin III activity or antigen levels decreased from 165 cases to 56. Overall, ordering behavior shifted to more guideline-supported indications.

Conclusions

This QI initiative demonstrated that combining EMR modifications with targeted provider education significantly improves the appropriate use of hypercoagulable testing. Interventions of this kind can reduce low-value testing, promote guideline adherence, and support more effective clinical decision-making.

## Introduction

Venous thromboembolism (VTE) remains a significant cause of morbidity and mortality, with more than one-third of annual cases related to recent hospitalization. Many of these events occur after discharge. VTE is the fifth most common cause of unplanned hospital readmission overall, and the third most common in patients undergoing total hip or knee replacement. Cancer and its treatments contribute to approximately 20% of VTE cases, and in about one-quarter of pulmonary embolism cases, sudden death is the first clinical presentation [1-4].

Routine hypercoagulable testing in unselected patients with VTE is not recommended. Most guidelines support testing only in specific populations, typically patients under the age of 45 years, those with recurrent thrombosis, individuals with thrombosis in unusual venous sites (e.g., portal, hepatic, mesenteric, or cerebral veins), and those with a family history of early-onset VTE. In such cases, evaluation may include testing for inherited thrombophilia, antiphospholipid syndrome, and, in select scenarios, *JAK2* mutations or paroxysmal nocturnal hemoglobinuria. While such testing can identify biologic risk factors and guide genetic counseling, it rarely alters long-term anticoagulation management or improves clinical outcomes. Moreover, its predictive value for recurrence is limited in unselected patients [5-8].

Despite its limited clinical utility in many inpatient settings, hypercoagulable testing is frequently overused. Ordering hypercoagulable workup during acute thrombotic events or while patients are receiving anticoagulation may yield misleading results and contribute to unnecessary healthcare expenditures [5,9].

The goal of this quality improvement (QI) initiative was to enhance provider understanding and appropriate utilization of hypercoagulable workups by implementing electronic medical record (EMR) changes and delivering targeted education to physicians. A specific focus was placed on reducing inpatient ordering of protein C, protein S, and antithrombin III activity/antigen levels, which are frequently inaccurate in the setting of acute thrombosis and recent anticoagulant exposure.

## Materials and methods

Before this QI initiative, the thrombosis panel available within our network included the following tests: lupus anticoagulant, cardiolipin antibodies, beta-2 glycoprotein antibodies, protein C activity, protein S activity, protein S antigen, and antithrombin III activity. Several of these tests are not recommended in patients with acute thrombosis or those receiving active or recent anticoagulation [6]. The previous order set did not provide a breakdown of individual components, and many providers were unaware of what tests were being ordered until results were reported. Additionally, in 2023, Factor V Leiden mutation and prothrombin gene mutation were removed from the thrombosis panel in our network and had to be ordered individually. However, this change was not widely communicated, and many providers were unaware of it.

We conducted a retrospective chart review of patients across all campuses within the St. Luke’s University Health Network who utilized the “Thrombosis Panel” from January 2024 to April 2024. To obtain pre-intervention data, we assessed the utilization patterns of thrombosis panels ordered during this period. Demographic data collected included patient age, gender, hospital site, encounter setting (inpatient vs. outpatient), and the ordering department. For each case, the clinical indication for the hypercoagulable workup was reviewed and categorized. Appropriateness of testing was determined based on documented clinical context and established guidelines, classifying each order as either appropriate or inappropriate.

As part of the intervention phase, the “Thrombosis Panel” was removed from the inpatient ordering system in Epic, with assistance from the IT department. The IT team collaborated with clinical leadership to disable the default panel and build a new ordering workflow. Providers were instead required to order individual components as clinically indicated and specify a justification based on the clinical scenario. The order entry workflow included a dropdown menu allowing providers to select from predefined indications, which included the following: unprovoked VTE, recurrent VTE, family or personal history of thrombosis suggesting prophylactic evaluation, unusual thrombosis sites (e.g., portal, hepatic, mesenteric, splenic, or cerebral veins), and arterial thrombosis in patients under 50 years of age.

Additionally, an acknowledgement tab was implemented for protein C activity, protein S activity, and antithrombin III activity ordering in Epic, requiring providers to acknowledge that the “test only yields accurate results when the patient has been off anticoagulation for at least two weeks and is at least three months post-acute thrombosis.”

We also provided educational lectures for providers in internal medicine, family medicine, and emergency medicine. These sessions were led by a hematology-oncology fellow involved in the QI initiative. The lectures addressed appropriate clinical indications for hypercoagulable testing, the limitations of performing such tests during acute thrombosis or anticoagulation, and guideline-based recommendations to support more evidence-based ordering practices.

## Results

During the pre-intervention period (January to April 2024), a total of 262 thrombosis panels were ordered across the St. Luke’s University Health Network, 165 in the inpatient setting and 97 in the outpatient setting. A chart review revealed that 237 (90.5%) of 262 panels were ordered inappropriately, based on established clinical criteria. Only 25 (9.5%) panels were considered appropriate. Furthermore, only four cases resulted in the identification of a true hypercoagulable condition.

**Figure 1:**
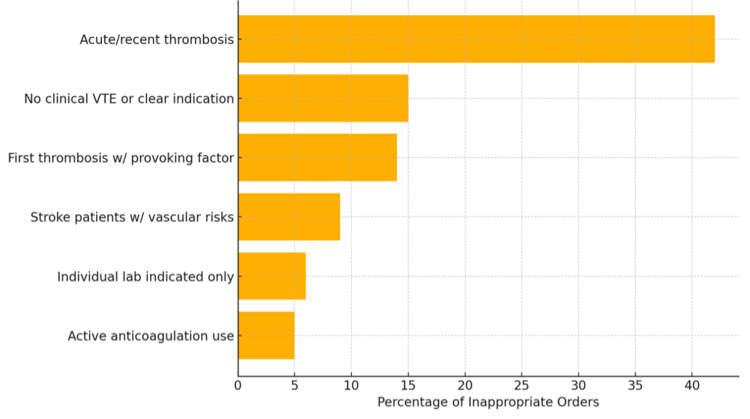
Indications for Thrombosis Panel orders (pre-intervention). VTE = venous thromboembolism

As illustrated in Figure 1, the leading cause of inappropriate testing was the ordering of the “Thrombosis Panel” during acute or recent thrombotic events (42%), when laboratory results are often unreliable, and guidelines advise against testing [6]. Other reasons included the first episode of thrombosis in patients with clear provoking factor(s) (14%), active anticoagulation use (5%), hypercoagulable workup ordered in stroke patients with obvious vascular risk factors and before the completion of routine stroke workup (9%), instances where only a specific individual lab was indicated, not the full panel (6%), and no clinical VTE or clear indication for hypercoagulable workup (15%).

**Figure 2:**
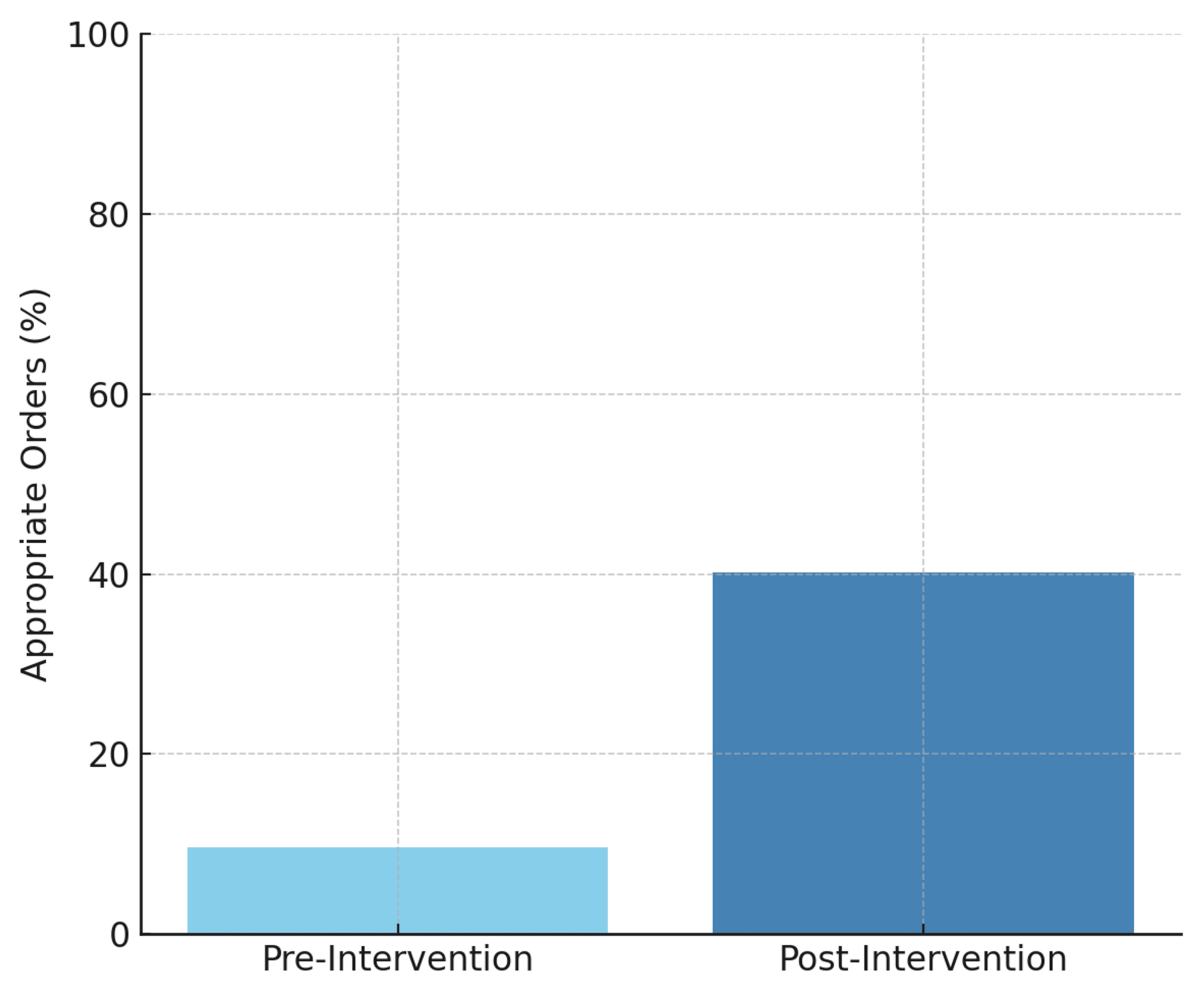
Inpatient hypercoagulable workup appropriateness and protein C/S and antithrombin III ordering.

Post-intervention data were collected from December 2024 to April 2025. Following the implementation of EMR changes and education sessions for the providers across the aforementioned departments, the appropriateness of hypercoagulable workup in the inpatient setting improved significantly from 9.7% in the pre-intervention phase to 40.2% post-intervention (Figure 2).

**Figure 3:**
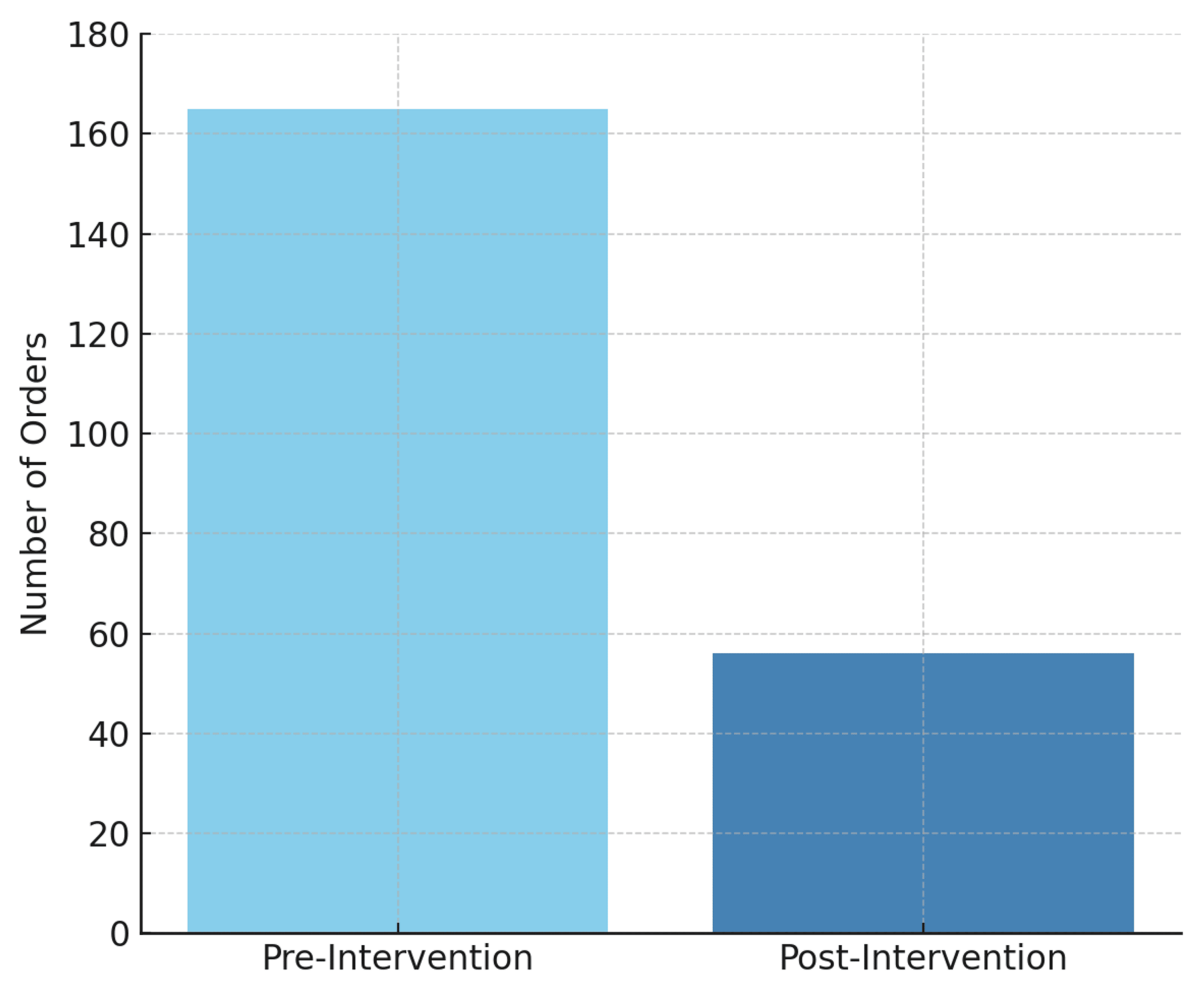
Protein C/S and antithrombin III ordered.

Importantly, the ordering of protein C, protein S, and antithrombin III assays, which are unreliable during acute thrombosis or in patients on anticoagulation, decreased from 165 cases pre-intervention to 56 cases post-intervention in the inpatient setting (Figure 3), indicating improved adherence to evidence-based recommendations.

## Discussion

The goal of this QI initiative was to enhance the understanding and clinical use of hypercoagulable workups by implementing EMR changes and delivering targeted education to providers. A specific focus was to reduce inpatient ordering of protein C, protein S, and antithrombin III levels, which are often rendered inaccurate by acute thrombosis or recent anticoagulation [6,7].

Routine hypercoagulable testing in unselected patients with VTE is not recommended and may lead to unnecessary downstream testing and costs [7,10]. Our findings confirm that the majority of pre-intervention testing was inappropriate, driven by limited awareness of appropriate indications and a lack of transparency regarding the panel’s components. The reduction in inappropriate testing observed post-intervention reflects improved provider awareness and more intentional ordering, likely influenced by the requirement to select a clinical indication and the shift toward ordering individual labs based on clinical context.

These interventions led to a substantial improvement in appropriate hypercoagulable workup ordering across a multi-campus hospital network. Following implementation, appropriate testing increased from 9.7% to 40.2%. Concurrently, inappropriate inpatient ordering of protein C, protein S, and/or antithrombin III activity or antigen levels decreased from 165 cases pre-intervention to 56 cases post-intervention. These results suggest that combining targeted education with EMR modifications can effectively reduce overutilization and promote guideline recommendations testing. Similar findings have been reported in previous QI initiatives aimed at optimizing thrombophilia test utilization [9].

This QI initiative has several limitations. First, as a retrospective chart review, it relied on the accuracy and completeness of documentation to assess appropriateness. Second, we did not measure cost-related outcomes associated with hypercoagulable test overutilization. Third, we did not include formal statistical testing such as p-values or confidence intervals; therefore, while observed pre- and post-intervention trends suggest improvement, these findings should be interpreted with caution regarding statistical significance. Finally, as long-term follow-up data were not collected, the durability of the intervention’s impact remains unknown [10-12].

Future directions include incorporating a standardized smart set for hypercoagulable workups in the ambulatory setting and expanding interventions to outpatient clinics and subspecialty services. Continued provider education will also be essential to reinforce appropriate testing indications and improve clinical decision-making around hypercoagulable evaluations [13-15].

## Conclusions

This QI initiative successfully improved the appropriateness of hypercoagulable workup ordering through a combination of EMR modifications and targeted provider education. The intervention significantly reduced inappropriate inpatient testing, particularly the overuse of protein C, protein S, and antithrombin III assays during acute thrombotic events or anticoagulation. These findings underscore the importance of aligning clinical decision-making with evidence-based guidelines. Continued reinforcement through education and EMR-based interventions will be important to sustaining and expanding these improvements.
